# Surgical Specialty Outcome Differences for Major Spinal Procedures in Low-Acuity Patients

**DOI:** 10.1177/21925682241288500

**Published:** 2024-10-01

**Authors:** Anthony Price, Christopher File, Alvin LeBlanc, Nathan Fredricks, Rylie Ju, Nathan Pratt, Rishi Lall, Daniel Jupiter

**Affiliations:** 1 John Sealy School of Medicine, 12338 The University of Texas Medical Branch at Galveston, Galveston, TX, USA; 2 Department of Neurosurgery, 12338 The University of Texas Medical Branch at Galveston, Galveston, TX, USA; 3 Department of Orthopaedic Surgery and Rehabilitation, 12338 The University of Texas Medical Branch at Galveston, Galveston, TX, USA; 4 Department of Biostatistics and Data Science, 12338 The University of Texas Medical Branch at Galveston, Galveston, TX, USA

**Keywords:** low-acuity patients, national surgical quality improvement plan, spinal outcomes, specialty comparison, spinal surgery

## Abstract

**Study Design:**

Retrospective Cohort Study.

**Objectives:**

There is an ongoing debate as to the influence of specialty training on spine surgery. Alomari et al. indicated the influence of specialty on ACDF procedures. However, deeper analysis into other spine procedures and lower-acuity procedures has yet to occur. In this study, we aim to determine if the outcomes of the low American Society of Anesthesiologists (ASA) classification (ASA 1&2) patients undergoing spine surgery vary based on whether the operating surgeon was an orthopedic surgeon or a neurosurgeon.

**Methods:**

The NSQIP databases from 2015 to 2021 were queried based on the CPT code for nine common spine procedures. Indicators of surgical course and successful outcomes were documented and compared between specialties.

**Results:**

Neurosurgeons had minimally shorter operative times in the ASA 1&2 combined classification (ASA-C) group for cervical, lumbar, and combined spinal procedural groups. Neurosurgeons had a slightly lower percentage of perioperative transfusions in select ASA-C classes. Orthopedic surgeons had shorter lengths of stay for the cervical groups in ASA-C and ASA-1 classes (ASA-1). However, many specialty differences found in spine patients become less pronounced when considering only ASA-1 patients. Finally, postoperative complication outcomes and re-admission were similar between orthopedic and neurological surgeons in all cases.

**Conclusions:**

These results, while statistically significant, are very likely clinically insignificant. They demonstrate that both orthopedic surgeons and neurosurgeons perform spinal surgery exceedingly safely with similarly low complication rates. This lays the groundwork for future exploration and benchmarking of performance in spine surgeries across neurosurgery and orthopedics.

## Introduction

Spinal surgery encompasses a broad spectrum of diagnoses, procedure types, anatomical locations, and patient outcomes. Common spine surgery types include decompressive laminectomies, discectomies, instrumented fusion, deformity correction, or combinations thereof, and are typically performed by neurosurgeons or orthopedic surgeons. Detailed analyses comparing specialty-specific spinal procedure outcomes are still limited.^1-5^ In many studies, the effect size was small and likely clinically insignificant. Other related studies supported few statistically significant differences and, when significant, were unlikely to have an impact on clinical practice.^2,6^ Recently, Alomari et al. revealed that neurosurgeons perform about three times as many anterior cervical discectomies and fusions (ACDFs) as orthopedic surgeons.^1^ They also showed that statistically, neurosurgeons achieved shorter hospital stays, lower perioperative blood transfusions, lower rates of sepsis, and operated on patient populations with more preoperative comorbidities; however, neurosurgeons did show longer operative times.^1^ A study comparing 90-day outcomes of elective 1 and 2-level posterior lumbar fusions between the specialties indicated similar outcomes, except for increased wound complications and decreased dural tears when surgery is conducted by neurosurgeons.^2^ Similarly, retrospective analysis of anterior lumbar fusions between specialties did not yield significant differences, aside from a higher risk for urinary tract infections and 30-day reoperation in the neurosurgery group.^7^ Other studies have also supported small differences and mostly have not seen significant outcome differences between the two specialties.^2,6^ McCutcheon and colleagues presented data displaying that orthopedic patients have higher rates of perioperative blood transfusions after matching for various factors.^2^ McCutcheon and other past studies focused on high-acuity patients or combined ASA classifications (ASA class 2+ patients)^1,2^ showing that high-acuity patients are more likely to be treated by neurosurgeons. While these studies have provided valuable insights into statistical specialty-specific differences, there remains a gap in the literature regarding the impact of surgeon specialty on patient outcomes in lower acuity cases.

Documentation of post-surgical outcomes for all surgical procedures has improved over recent decades partly due to the continuously expanding use of the National Surgical Quality Improvement Plan (NSQIP).^8^ Created in the 1980s and currently managed by the American College of Surgeons, this database provides commonly accepted metrics for postoperative outcomes. As of today, more than 600 hospitals and over 65 Collaboratives participate in the quality metric reporting conducted by the NSQIP.^8,9^ The cost of spinal procedures often imposes a significant burden on both the healthcare system and the patient. As indicated in the report on Operating Room Procedures During Inpatient Stays in U.S. Hospitals in 2018, hospital stays involving spinal fusion accounted for the highest total expenditure for any procedure type, totaling $14.1 billion.^10^ Surgical interventions that yield improved outcomes are crucial for alleviating this cost burden.^10^ Understanding the differential outcomes between orthopedic and neurological approaches not only aids referring physicians in making informed clinical decisions but also opens avenues for understanding and collaboration between the specialties to optimize patient care. Given the considerable overlap in surgical competencies, cost burden, inconclusive historical results, and lack of outcome results on collective spinal operations, additional data comparing neurosurgical and orthopedic approaches to spine surgery is necessary.

In this study, we aim to determine if the outcomes and characteristics of low-ASA classification patients undergoing surgical interventions vary based on whether the surgeon operating was trained in orthopedics or neurosurgery. We obtained deidentified patient data on spinal surgical interventions, stratified according to the surgeon’s specialty. Indicators of the surgical course and outcomes were documented and compared between specialties at a patient population level.

## Methods

### Data Acquisition and Variables Collected

The NSQIP databases^11^ from 2015 to 2021 (the latest year available at the time of writing) were queried for 9 of the most common spinal procedure CPT codes (22551, 22554, 63075, 22600, 22633, 22612, 22630, 63030, 63047) reported on by other studies^12^: these represent anterior cervical discectomy and fusion (ACDF), lumbar discectomies, posterior cervical and lumbar fusion surgery, and decompressive laminectomy. Full descriptions of each CPT code are shown in Table 1. Patients were included if one of these CPT codes appeared as the primary operative code.

**Table 1.** CPT Codes of Common Spinal Surgeries: The 9 Most Common Spinal Procedures With Their CPT Code, the Short Descriptive Name, and the Longer Descriptive Procedure Explanation.

CPT Codes and Description of Procedures		
CPT Code	Short Description	Long Description
22551	Anterior Cervical Discectomy and Fusion (ACDF)	Anterior or anterolateral approach technique arthrodesis Procedures on the spine (Vertebral Column)
22554	Neck spine fusion	Arthrodesis, anterior interbody technique, including minimal discectomy to prepare interspace (other than for decompression)
63075	Neck spine disk surgery	Discectomy, anterior, with decompression of spinal cord and/or nerve root(s), including osteophytectomy
22600	Neck spine fusion (posterior cervical fusion)	Arthrodesis, posterior or posterolateral technique, single level
22633	Lumbar spine fusion combined (TLIF)	Arthrodesis, combined posterior or posterolateral technique with posterior interbody technique including laminectomy and/or discectomy sufficient to prepare interspace (other than for decompression), single interspace and segment
22612	Lumbar spine fusion	Arthrodesis, posterior or posterolateral technique, single level
22630	Lumbar spine fusion with laminectomy or discectomy	Arthrodesis, posterior interbody technique, including laminectomy and/or discectomy to prepare interspace (other than for decompression), single interspace
63030	Low back disk surgery	Laminotomy (hemilaminectomy), with decompression of nerve root(s), including partial facetectomy, foraminotomy and/or excision of herniated intervertebral disc
63047	Removal of spinal lamina (L-laminectomy)	Laminectomy, facetectomy and foraminotomy (unilateral or bilateral with decompression of spinal cord, cauda equina and/or nerve root[s], [eg, spinal or lateral recess stenosis]), single vertebral segment

Variables collected were sex, race, ethnicity (Hispanic & Non-Hispanic), American Society of Anesthesiologists (ASA) class (only 1 and 2),^13^ diabetes, history of COPD, history of smoking, history of use of hypertension medication, history of steroid use, history of bleeding disorders, and age (Table 2). Surgical specialties were restricted to neurosurgery and orthopedic surgery. The outcome variables evaluated were operation time, length of hospital stay, perioperative transfusions (red blood cell transfusions within the first 72 h of surgery start time), the occurrence of wound disruptions, deep vein thrombosis (DVT), or pulmonary embolisms, and readmission (within 30 days of discharge). The NSQIP database does not provide surgeon or hospital identifiers, so more discrete details on this information could not be gathered. Other risk factor scores like the Charlson Comorbidity Index are also unavailable in the NSQIP.

**Table 2.** Descriptive Statistics of Combined Spinal Procedure Patients: Demographic, Preoperative Comorbidity, and Surgical Outcome Data of the ASA-C Class Patient Population.

Descriptive Analysis of Combined Spinal Procedure Patients		
	Number	Percentage (%)
All Patients	150,469	
Male	78,588	52.23
Neurosurgery Cases	100,696	66.92
Orthopedic Surgery Cases	49,773	33.08
Race (n = 134,619)		
White	116,662	86.66
Black or African American	11,799	8.77
Asian	4567	3.39
Other	871	.65
American Indian or Alaska Native	720	.54
Missing	15,850	
Ethnicity (n = 136,616)		
Non-Hispanic	126,420	92.54
Hispanic	10,196	7.46
Missing	15,850	
ASA Classification		
1-No Disturb	11,934	7.93
2-Mild Disturb	138,535	92.07
History of severe COPD	1696	1.13
Diabetes mellitus with oral agents or insulin (n = 147,749)	9910	6.71
Missing	2720	
Current smoker within one year	28,664	19.05
Hypertension requiring medication	52,965	35.20
Immunosuppressive Therapy	3934	2.61
Bleeding disorders	621	.41
Wound	210	.14
DVT	507	.34
PE	372	.25
Readmission (within 30 days)	4217	2.80

Abbreviations: ASA-C, ASA combined classes 1 and 2, COPD, chronic obstructive pulmonary disease, DVT, deep vein thrombosis, PE, pulmonary embolism.

### Data Stratification

**Figure 1.**
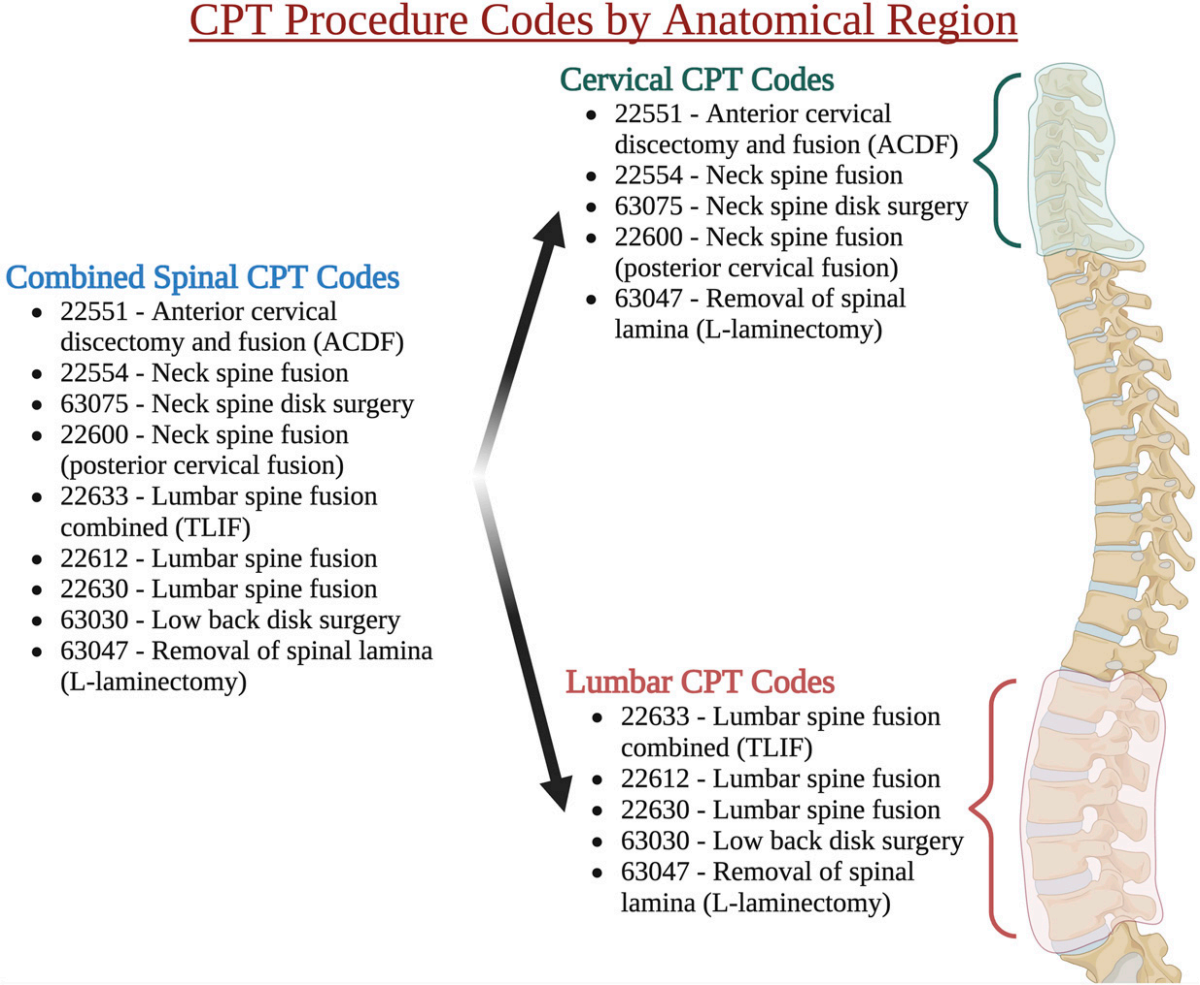
CPT procedure codes by anatomical region: reported as CPT number - procedure name. CPT, current procedural terminology, ACDF, anterior cervical discectomy and fusion, created with biorender.

Patients were stratified into three spinal procedure groups by anatomical clusters (cervical, lumbar, and combined spinal procedure codes) since spinal operations are rarely completed as single CPT codes but rather as clusters of procedures (eg, laminectomy plus fusion). The combined spinal group consists of CPT codes 22551, 22554, 63075, 22600, 22633, 22612, 22630, 63030, and 63047. The cervical spinal group consists of CPT codes 22551, 22554, 63075, 22600, and 63047. The lumbar spinal group consists of CPT codes 22633, 22612, 22630, 63030, and 63047. These groupings can be found in Figure 1. The CPT codes for 63047, laminectomy, are recorded in both anatomical locations, so they were included in both anatomical location groups.

This data was then further stratified by ASA classification: a combined ASA-1&2 group (ASA-C), and an ASA-1 group (ASA-1). ASA classifications 1 and 2 indicate a healthy patient or a patient with non-life-threatening comorbidities, respectively.^13^ These ASA classes were selected to minimize any potential contributing factors of more severe systemic disease and higher levels of comorbidities that may occur in ASA classes ≥ 3. The other demographic and preoperative factors above were chosen for their potential influence on the hospital course, discharge, and postoperative complications, and many were also included in past studies.^1^

Initially, the study aimed to analyze and present ASA-1, ASA-2, and ASA-C individually between the two specialties. However, given the majority of patients fell under the ASA-2 classification (>90% for all given surgery comparisons) the presentation of only the ASA-2 classification is similar to the ASA-C group, so we chose to only present ASA-1 and ASA-C for comprehensive analysis.

### Statistical Analysis

Continuous and categorical variables were described using means (standard deviations) and frequencies (proportions), respectively. For each categorical outcome, association with independent variables was assessed using t-tests and chi-squared tests, for continuous and categorical independent variables, respectively. For each continuous outcome, association with independent variables was assessed using correlation coefficient and *t*-test/ANOVA, for continuous and categorical independent variables, respectively. The surgical specialty was included in all models. The analysis was repeated restricting the dataset to ASA-1 patients, to those with cervical procedures, and to those with lumbar procedures.

All analyses were carried out using R version 4.3.1. and RStudio Build 421.^14^

## Results

### Combined Spinal Procedure Outcomes

**Figure 2.**
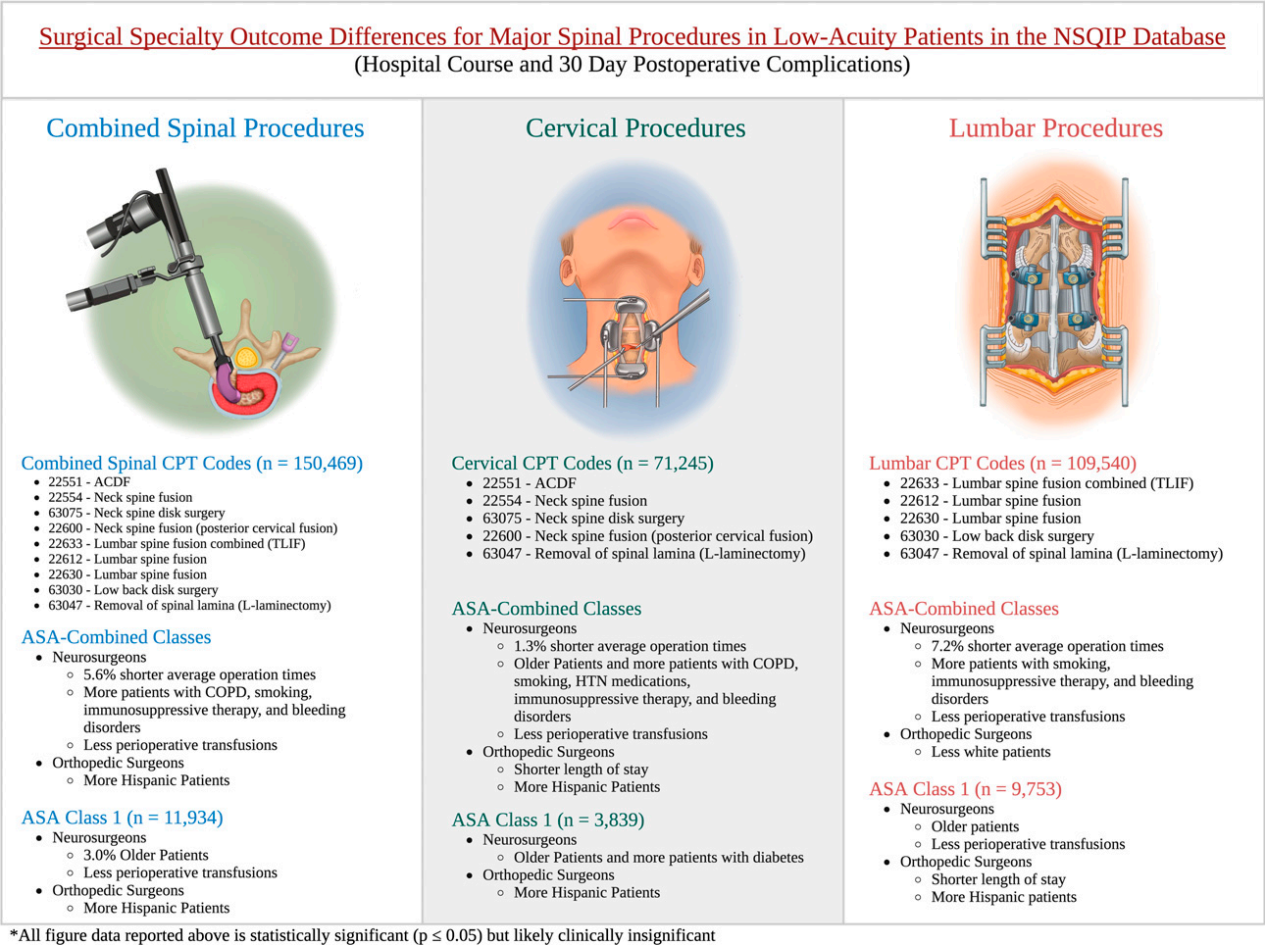
Surgical specialty outcomes by ASA-classification and anatomical region: generalized differences regarding the demographics, preoperative comorbidities, and surgical outcomes of ASA-C and ASA-1 patient populations between neurosurgery and orthopedic surgery by surgical region. ASA-C, ASA combined classes 1 and 2, ASA-1, ASA-1 class, CPT, current procedural terminology, ACDF, anterior cervical discectomy and fusion, COPD, chronic obstructive pulmonary disease, HTN, hypertension, created with biorender.

Neurosurgery patients were slightly older on average (41.8 years ± 13.0 vs 40.6 years ± 13.0) in the ASA-1 group (*P* < .01) (Table 3). A higher percentage of orthopedic surgery patients were Hispanic (9.9% vs 8.0%) in the ASA-1 group (*P* < .01). A higher percentage of neurosurgery patients were white (86.7% vs 84.6%) in the ASA-1 group (*P* < .01) (Figure 2). A higher percentage of neurosurgery patients were on immunosuppressive therapy (1.4% vs .9%) in the ASA -1 group (*P* = .03). Orthopedic surgery patients had a slightly longer operation time (136.9 min vs 129.7 min) in the ASA-C group (*P* < .01). Neurosurgery patients had a slightly longer length of stay (1.2 days vs 1.0 days) in the ASA-1 group (*P* < .01). Orthopedic surgery patients had a slightly higher average of perioperative transfusions (.028 vs .016) compared to neurosurgery patients in the ASA-C group (*P* < .01).

**Table 3.** Combined Spinal Outcomes by Specialty: Comparison of Demographics, Preoperative Comorbidities, and Outcome Measures for ASA-C and ASA-1 Patients Between Neurosurgery and Orthopedic Surgery for all Spinal Procedures. The Data is Reported as Either: Mean ± Standard Deviation or as Number (Percentage). Data With Significant *P*-Values are Bolded. Operation Time is Reported in Minutes and Length of Stay is Reported in Days.

Combined Spinal Outcomes by Specialty						
	Combined Spinal Cases - ASA-C (n = 150,469)			Combined Spinal Cases - ASA-1 (n = 11,934)		
	Neurosurgery	Orthopedic Surgery		Neurosurgery	Orthopedic Surgery	
Outcome Measures	n = 100,696 (66.9%)	n = 49,773 (33.1%)	*P* value	n = 7823 (65.6%)	n = 4111 (34.4%)	*P* value
Demographics						
Age	53.8 ± 14.4	53.8 ± 14.5	.99	**41.8** ± 13.0	40.6 ± 13.0	**<.01**
Hispanic	6708 (7.2)	3488 (**7.9**)	**<.01**	541 (8.0)	338 (**9.9**)	**<.01**
Male	52,512 (52.2)	26,076 (52.4)	.38	4712 (60.2)	2541 (61.8)	.09
White	79,009 (**87.4**)	37,653 (85.2)	**<.01**	5722 (**86.7**)	2856 (84.6)	**<.01**
Preoperative Comorbidities						
History of severe COPD	1177 (**1.2**)	519 (1.0)	**.03**	12 (.2)	2 (.1)	.11
History of Diabetes	6593 (6.7)	3317 (6.8)	.40	38 (.5)	18 (.4)	.72
Current Smoker within 1 year	20,030 (**19.9**)	8634 (17.3)	**<.01**	759 (9.7)	368 (9.0)	.18
Hypertension requiring medication	35,517 (35.3)	17,448 (35.1)	.41	210 (2.7)	114 (2.8)	.78
Immunosuppressive therapy	2770 (**2.8**)	1164 (2.3)	**<.01**	107 (**1.4**)	37 (.9)	**.03**
History of Bleeding Disorders	459 (**.5**)	162 (.3)	**<.01**	7 (.1)	1 (.0)	.19
Hospital Course & Discharge						
Operation Time	129.7 ± 82.3	**136.9** ± 87.7	**<.01**	106.1 ± 69.0	106.5 ± 69.4	.78
Length of Stay	1.8 ± 2.5	1.8 ± 2.5	.32	**1.2** ± 3.6	1.0 ± 4.8	**<.01**
Readmission (within 30 days)	2828 (2.8)	1389 (2.8)	.84	124 (1.6)	55 (1.3)	.29
Medical Complications						
Wound Dehiscence Occurrences	151 (.2)	59 (.1)	.13	3 (.04)	2 (.05)	.79
Deep Vein Thrombosis Occurrences	337 (.3)	170 (.3)	.83	14 (.2)	8 (.2)	.85
Pulmonary Embolism Occurrences	237 (.2)	135 (.3)	.19	12 (.2)	5 (.1)	.66
Average Perioperative Transfusions	.016 ± .127	**.028** ± .165	**<.01**	.005 ± .074	**.009** ± .096	**.03**

Abbreviations: n, number, ASA-C, ASA combined classes 1 and 2, ASA-1, ASA-1 class, COPD, chronic obstructive pulmonary disease, *P* ≤ .05.

### Cervical Procedure Outcomes

Neurosurgery patients were slightly older on average (55.9 years ± 12.9 vs 55.4 years ± 13.0) in the ASA-C group (*P* < .01) (Table 4). A higher percentage of orthopedic surgery patients were Hispanic (7.5% vs 6.7%) in the ASA-C group (*P* < .01). A higher percentage of neurosurgery patients were White (86.9% vs 84.5%) in the ASA-C group (*P* < .01). A higher percentage of neurosurgery patients were current smokers (20.6% vs 18.4%) in the ASA-C group (*P* < .01). A higher percentage of neurosurgery patients required medication for hypertension (38.2% vs 36.9%) in the ASA-C group (*P* < .01). A higher percentage of neurosurgery patients were on immunosuppressive therapy (2.8% vs 2.4%) in the ASA-C group (*P* = .01). A higher percentage of neurosurgery patients had a history of bleeding disorders (.5% vs .4%) in the ASA-C group (*P* = .01). Orthopedic surgery patients had a slightly longer operation time (126.2 min vs 124.6 min) in the ASA-C group (*P* < .01). Neurosurgery patients had a slightly longer length of stay (1.62 days vs 1.54 days) in the ASA-C group (*P* < .01). Orthopedic surgery patients had a slightly higher average of perioperative transfusions (.013 vs .007) in the ASA-C group (*P* < .01).

**Table 4.** Cervical Spine Procedure Outcomes by Specialty: Comparison of Demographics, Preoperative Comorbidities, and Outcome Measures for ASA-C and ASA-1 Patients Between Neurosurgery and Orthopedic Surgery for Only Cervical Procedures. The Data is Reported as Either: Mean ± Standard Deviation or as Number (Percentage). Data With Significant *P*-Values are Bolded. Nearly Significant *P*-Values are Highlighted. Operation Time is Reported in Minutes and Length of Stay is Reported in Days.

Cervical Procedure Outcomes by Specialty						
	Cervical Cases - ASA-C (n = 71,245)			Cervical Cases - ASA-1 (n = 3839)		
	Neurosurgery	Orthopedic Surgery		Neurosurgery	Orthopedic Surgery	
Outcome Measures	n = 49,086 (68.9%)	n = 22,159 (31.1%)	*P* value	n = 2575 (67.1%)	n = 1264 (32.9%)	*P* value
Demographics						
Age	**55.9** ± 13.2	55.4 ± 13.2	**<.01**	**46.4** ± 12.9	44.9 ± 13.0	**<.01**
Hispanic	3058 (6.7)	1526 (**7.5**)	**<.01**	169 (7.3)	117 (**10.8**)	**<.01**
Male	25,601 (52.1)	11,728 (52.9)	.06	1477 (57.4)	731 (57.8)	.78
White	38,910 (**86.9**)	16,988 (84.5)	**<.01**	1942 (**86.5**)	869 (82.9)	**.01**
Preoperative Comorbidities						
History of severe COPD	672 (**1.37**)	255 (1.2)	**.017**	7 (.3)	0 (.0)	.06
History of Diabetes	3461 (7.2)	1587 (7.3)	.63	21 (**.008**)	3 (.002)	**.03**
Current Smoker within 1 year	10,101 (**20.6**)	4078 (18.4)	**<.01**	258 (10.0)	129 (10.2)	.86
Hypertension requiring medication	18,743 (**38.2**)	8176 (36.9)	**<.01**	107 (4.2)	40 (3.2)	.13
Immunosuppressive therapy	1330 (**2.7**)	527 (2.4)	**.01**	36 (1.4)	11 (.9)	.16
History of Bleeding Disorders	244 (**.5**)	80 (.4)	**.01**	3 (.12)	0 (.0)	.23
Hospital Course & Discharge						
Operation Time	124.6 ± 70.4	**126.2** ± 71.6	**<.01**	116.0 ± 66.4	114.9 ± 70.0	.64
Length of Stay	**1.62** ± 3.6	1.54 ± 4.0	**<.01**	1.39 ± 2.1	1.33 ± 2.4	.42
Readmission (within 30 days)	1306 (2.7)	580 (2.6)	.74	33 (1.3)	15 (1.2)	.80
Medical Complications						
Wound Dehiscence Occurrences	69 (.1)	31 (.1)	.98	∅	∅	NA
Deep Vein Thrombosis Occurrences	140 (.3)	61 (.3)	.82	4 (.2)	2 (.2)	.98
Pulmonary Embolism Occurrences	100 (.2)	45 (.2)	.99	6 (.2)	0 (.0)	.09
Average Perioperative Transfusions	.007 ± .085	**.013** ± .114	**<.01**	.003 ± .052	.008 ± .089	.054

Abbreviations: n, number, ASA-C, ASA combined classes 1 and 2, ASA-1, ASA-1 class, COPD, chronic obstructive pulmonary disease, ∅, empty set, NA, not available, *P* ≤ .05.

### Lumbar Procedure Outcomes

Neurosurgery patients were slightly older on average (41.0 years ± 13.3 vs 39.9 years ± 13.3) in the ASA-1 group (*P* < .01) (Table 5). A higher percentage of orthopedic surgery patients were Hispanic (9.5% vs 8.1%) in the ASA-1 group (*P* = .034). A higher percentage of neurosurgery patients were White (86.8% vs 85.6%) in the ASA-1 group (*P* = .002). Neurosurgery patients had a slightly longer length of stay (1.26 days vs 1.09 days) in the ASA-1 group (*P* = .001). Orthopedic surgery patients had a slightly higher average of perioperative transfusions (.01 vs .007) in the ASA-1 group (*P* = .05).

**Table 5.** Lumbar Spine Procedure Outcomes by Specialty: Comparison of Demographics, Preoperative Comorbidities, and Outcome Measures for ASA-C and ASA-1 Patients Between Neurosurgery and Orthopedic Surgery for Only Lumbar Procedures. The Data is Reported as Either: Mean ± Standard Deviation or as Number (Percentage). Data With Significant *P*-Values are Bolded. Operation Time is Reported in Minutes and Length of Stay is Reported in Days.

Lumbar Procedure Outcomes by Specialty						
	Lumbar Cases - ASA-C (n = 109,540)			Lumbar Cases - ASA-1 (n = 9753)		
	Neurosurgery	Orthopedic Surgery		Neurosurgery	Orthopedic Surgery	
Outcome Measures	n = 71,990 (65.7%)	n = 37,550; (34.3%)	*P* value	n = 6304 (64.6%)	n = 3449 (35.4%)	*P* value
Demographics						
Age	54.1 ± 15.3	54.1 ± 15.4	.77	**41.0** ± 13.3	39.9 ± 13.3	**<.01**
Hispanic	4942 (7.5)	2593 (7.9)	.056	442 (8.1)	271 (**9.5**)	**.03**
Male	38,228 (53.1)	19,968 (53.2)	.81	3890 (61.7)	2173 (63.0)	.21
White	56,574 (**88.1**)	28,408 (85.8)	**<.01**	4574 (**86.8**)	2421 (85.6)	**<.01**
Preoperative Comorbidities						
History of severe COPD	782 (1.1)	374 (1.0)	.165	9 (.1)	2 (.1)	.23
History of Diabetes	4839 (6.9)	2575 (7.0)	.415	23 (.4)	16 (.5)	.46
Current Smoker within 1 year	13,202 (**18.3**)	6010 (16.0)	**<.01**	589 (9.3)	286 (8.3)	.08
Hypertension requiring medication	25,976 (36.0)	13,498 (36.0)	.66	152 (2.4)	94 (2.7)	.34
Immunosuppressive therapy	2016 (**2.8**)	907 (2.4)	**<.01**	85 (1.4)	32 (.9)	.07
History of Bleeding Disorders	334 (**.5**)	127 (.3)	**<.01**	6 (.10)	1 (.03)	.24
Hospital Course & Discharge						
Operation Time	130.3 ± 87.2	**139.7** ± 92.9	**<.01**	116.0 ± 66.4	114.9 ± 69.0	.64
Length of Stay	1.8 ± 3.6	1.8 ± 3.7	.131	**1.3** ± 2.5	1.1 ± 2.4	**<.01**
Readmission (within 30 days)	2196 (3.0)	1141 (3.0)	.914	110 (**1.8**)	51 (1.5)	.32
Medical Complications						
Wound Dehiscence Occurrences	120 (.2)	45 (.1)	.058	3 (.05)	2 (.06)	.83
Deep Vein Thrombosis Occurrences	270 (.4)	149 (.4)	.58	12 (.2)	8 (.2)	.66
Pulmonary Embolism Occurrences	188 (.3)	115 (.3)	.177	8 (.1)	5 (.1)	.82
Average Perioperative Transfusions	.022 ± .1	**.036** ± .2	**<.01**	.007 ± .08	**.010** ± .1	**.05**

Abbreviations: n, number, ASA-C, ASA combined classes 1 and 2, ASA-1, ASA-1 class, COPD, chronic obstructive pulmonary disease, *P* ≤ .05.

## Discussion

Comparing surgical specialties regarding spine outcomes continues to be challenging due to the varying metrics that define success. Commonly held qualities of a “successful” surgery, such as shorter operation times and fewer postoperative complications, have been previously used for comparison of orthopedic and neurological spine surgery.^1,2,15^ The observed differences are often clinically negligible. This analysis reaffirms many previous findings, highlighting statistically significant yet likely clinically insignificant differences between the specialties. Regardless of the training path, these outcomes represent real patients and the relief provided by both orthopedic and neurological surgeons. To date, there have been multiple database studies comparing orthopedic and neurosurgery outcomes for spine surgery, most of them (eg, McCutcheon^2^) propensity match the patients to minimize any potential confounding variables and compare outcomes within groups of essentially identical patients. Our focus was slightly different, intending to compare the patient outcomes for these two specialties at a larger, unfiltered population level. This allows us to discuss the potential impacts of confounding variables on the outcomes. Past analyses have also investigated specific surgical procedures; however, spinal operations are rarely completed as single CPT codes but rather as clusters of codes (eg, laminectomy plus arthrodesis plus segmental instrumentation) usually pertaining to specific anatomical regions. This necessitates reconsideration for reporting categorical spinal outcomes such as our proposed anatomical groups (cervical, lumbar, and combined spinal procedures) rather than singular procedure types so that more generalizable inferences can be made (Supplemental Material).

We identified consistent demographic and pre-morbid patterns among patients in the ASA-C class undergoing orthopedic and neurosurgical interventions. The focus on the ASA-C group allows us to discuss outcomes in patients that are a majority ASA-2. Neurosurgeons tended to operate on patients with a greater burden of comorbidities, a trend that held across anatomical subgroups. Specifically, cervical cases managed by neurosurgeons more often involved hypertensive patients. Conversely, the prevalence of COPD, diabetes, and hypertension were statistically comparable between the two surgical specialties in the lumbar region.

When comparing all spinal cases in this study, neurosurgeons had statistically significantly shorter operation times by an average of 7.2 min. This difference, notably, is in opposition to past studies that reveal shorter operative times for orthopedic surgeons. Lambrechts et al. found that orthopedic surgeons were finished, on average, 14.28 min faster (95% CI: 8.07, 20.49) while Alomari et al. found orthopedists were finishing earlier in both single and multi-level ACDFs.^1,15^ However, the difference shown in our analysis was no longer observable when inspecting just the healthiest ASA-1 cases in both the combined spinal and anatomically divided groups. This suggests that routine procedures on the healthiest patients are performed with similar speed. Additionally, neurosurgeon operative times were a statistically significant 9.4 minutes shorter on average for lumbar spine surgery. It is unclear if shorter lumbar procedural times represent differences in case mix, surgical technique, training, or surgical efficiency. Future studies may address this.

It is also important to consider the clinical impact of these differences. While statistically significant, differences in surgical times under 10 min are by no means clinically significant for operations that are hours long. Though neurosurgeon surgery times were, on average, less than 10 min longer, outcomes between specialties were indistinguishable for post-operative wound dehiscence, deep vein thrombosis, pulmonary embolism, and re-admission. These similarities in positive outcome measures were consistent across each anatomical location and patient class. Additionally, previous studies have not established consistent differences in patient-reported outcome measures between specialties, and while we have presented statistically different differences, the clinical implications are likely marginal at best.

One important factor to consider is how operative time may affect transfusion rates. In our analysis, neurosurgical cases required slightly fewer transfusions in cervical, lumbar, and combined cases. This difference remained when specifically addressing the ASA-1 class which was possibly driven by the larger quantities of transfusions in lumbar cases. The shortened operative times could be a contributing factor to this decrease in intraoperative bleeding, and thus the lower transfusion rate seen in neurosurgeon cases. That said, it is difficult to confirm that differences in operative time under 10 min would make a clinical impact on postoperative transfusion rates. For example, Alomari et al. and Lambrechts et al. both found neurosurgeons to have longer operative times but also fewer post-operative transfusions.^1,15^ This is more indicative of other factors at play besides small differences in operative time. It is possible that criteria for transfusion may vary between specialties and neurosurgeons may be more conservative in this regard.

Clinically, differences in transfusion rates offer a possible way to maximize patient safety and minimize healthcare costs. Because increased blood transfusions lead to higher perioperative morbidity, reductions in transfusion rates not only protect patients but also can save health systems financially.^16,17^ One major hospital reduced the use of blood products by 26.1% and saved over $2,200,000 in a single year.^17^ This highlights both the medical and financial benefits of reducing transfusion rates for colleagues in both neurosurgical and orthopedic specialties. While both specialties boast low transfusion rates for spine surgery, the goal should always be fewer if possible.

For postoperative stay, in all anatomical and ASA subgroups except the cervical ASA-1 and lumbar ASA-C cohorts, neurosurgery patients experienced longer stays compared to their orthopedic counterparts. It is plausible that orthopedic surgeons may systematically aim for earlier discharges, potentially emphasizing outpatient management as part of a care strategy. By emphasizing earlier discharge, patients can return to a more comfortable environment to both begin recovery as well as avoid major healthcare costs associated with inpatient stays. As for ASA-C cervical cases, the significantly longer hospital stays observed among neurosurgeons’ patients could be linked to a higher incidence of comorbidities, which may necessitate more extensive inpatient management and rehabilitation.

As with transfusion rates, reducing the length of stay poses significant opportunities for health systems to reduce the financial burdens of surgical intervention.^18-20^ While reducing the length of stay relies on many moving pieces, surgeons play a central role in postoperative planning. When surgeons implement programs such as at-home rehab programs or case management, patients are able to move to at-home recovery more quickly. In one New York study across all surgical patients in multiple hospitals, physicians found that length of stay-reducing measures could save over 10,000 inpatient days for surgical patients, by conservative estimates.^19^ Higher estimates found that as many as 25,000 inpatient days were prevented for surgical patients across the health system studied.^19^ When costs were accounted for, the study estimated that length of stay interventions saved between 4.2-6.3 million dollars over five years. Additionally, a large study with over 670,000 spine surgery patients found that a 1% increase in length of stay led to a .47% increase in cost.^20^ This demonstrates, on a large scale, how important it is to minimize length of stay. When considering the impact that these interventions can have, it becomes clear how important it is for spine surgeons to encourage patients to recover at home as soon as is reasonable and safe. This will help patients to save money, time, and has the potential to help reduce patient burdens on the larger healthcare ecosystem.

Discrepancies in the data may exist as compared to other data sets since we did not propensity score match cohorts. Significant factors, including demographics and comorbidities, could potentially influence the final observed differences in our study. To account for this, we controlled our data analysis based on ASA class, a system used to classify patients’ operative and perioperative risk according to their comorbidities. While the difference in these rates of comorbidities was small, these results showed significant differences between the specialties. The ASA-1 classification, despite being described as “normal, healthy patients” by the ASA, still contains patients with comorbidities. We observed comorbidity disparities in these patients between neurosurgeons and orthopedic surgeons performing spine surgery even in this lower ASA-1 classification. This could be contributing to the differences in outcomes seen in the specialties.

Another weakness of the study is the lack of evaluation of multiple levels operated in each case by not delineating in our analysis the additional spinal level CPT codes (eg, a one-level fusion vs a three-level fusion). Naturally, the number of levels operated affects the complexity of the case, perioperative blood loss, the likelihood of complications, and the potential operative outcome prognosis. McCutcheon et al. already explored the effect of multiple levels on outcomes with no significant difference found between specialties.^2^ We believe that the sheer volume of cases explored in this study (150,469 cases) and McCutcheon’s findings are enough to make this lack of data delineation negligible, but further studies should be done to help explore the effect of the number of levels on outcomes.

Given the above, our results should be considered preliminary and indicative of areas for further, more detailed investigation. Future studies should aim for more controlled and granular analyses to provide deeper insights into specialty-specific outcomes in spine surgery.

The authors plan to bring this information to the attention of the American Spine Registry (ASR) and the Neuropoint Alliance Quality Outcomes Database (QOD) Spine Registry. The ASR, a collaborative effort between the American Association of Neurological Surgeons (AANS) and the American Academy of Orthopaedic Surgeons (AAOS), was designed to create a more comprehensive, collaborative effort between specialties to improve patient care and spinal surgery.^21-24^ The QOD Spine Registry is the United States’ largest spinal registry.^25^ The above findings can be used to inform the ASR’s and QOD’s current data collection metrics and benchmarks of potential outcome differences that warrant deeper examination. The ASR and QOD already use similar systems of cervical and lumbar procedure stratification easing the incorporation of our performance measures. This study can also steer future projects led by the ASR and spinal surgeons to improve future clinical outcomes and improve standards of care.

Further, the findings of the study demonstrate the opportunities for continued collaboration between the two specialties to leverage the strengths observed in each field to improve patient care and health systems. Future research should prioritize cross-specialty collaborative investigations into the minutia of operative techniques and the decision-making processes employed by the observed specialties which underpin the marginal differences in operative statistics discovered by this study. Additionally, future studies focusing on minimally clinically significant measures can help to distinguish impactful areas of improvement between specialties. Overall, this research will be useful in driving essential initiatives, like those mentioned above, to foster shared learning and protocol development, particularly focusing on managing comorbidities and the optimization of operations times and transfusion practices. Additionally, this research advocates for the continued development and growth of robust and nuanced standardized data registries containing pertinent surgeon and hospital identifiers to allow for increased granularity and applicability in future studies. By doing so, we can aim to improve patient outcomes, reduce healthcare costs, and set new benchmarks for excellence in spinal surgery.

## Conclusion

The NSQIP database was examined to compare preoperative comorbidities and common metrics for successful outcomes in neurological and orthopedic spine. Overall, many operative characteristics and postoperative outcomes were statistically similar in almost all categories in low-acuity patients. However, while statistically significant, clinical significance is unlikely. This finding is further supported by isolating ASA-1 patients, which showed both fewer differences in each group as well as a smaller effect size. This preliminary study lays the groundwork for future collaboration, benchmarking of performance, and exploration in spine surgery across neurosurgery and orthopedics.

## Supplemental Material

Supplemental Material - Surgical Specialty Outcome Differences for Major Spinal Procedures in Low-Acuity Patients Supplemental Material for Surgical Specialty Outcome Differences for Major Spinal Procedures in Low-Acuity Patients by Anthony Price, Christopher File, Alvin LeBlanc, Nathan Fredricks, Rylie Ju, Nathan Pratt, Rishi Lall, and Daniel Jupiter in Global Spine Journal
